# Restricted *Pax3* Deletion within the Neural Tube Results in Congenital Hydrocephalus

**DOI:** 10.3390/jdb4010007

**Published:** 2016-02-01

**Authors:** Hong-Ming Zhou, Simon J. Conway

**Affiliations:** Herman B. Wells Center for Pediatric Research, Department of Pediatrics, Indiana University School of Medicine, Indianapolis, Indiana, IN 46202, USA; hongzhou@iu.edu

**Keywords:** mouse embryo, neural tube defects, Pax3, Pax7, congenital hydrocephalus, lineage mapping

## Abstract

Congenital hydrocephalus is a common birth-defect whose developmental origins are poorly understood. *Pax3-*null mutants show defects in myogenesis, neural tube closure, neural crest morphogenesis, and heart development that, consequently, results in embryonic lethality. Here we demonstrate that conditional deletion of the mouse *Pax3* transcription factor results in fully-penetrant congenital obstructive hydrocephalus. To identify the role of Pax3 during cranial development, we deleted *Pax3* within the neuroepithelium (via *Pax7^−Cre^*), in the neural crest (via *P0-Cre*), and in both the neuroepithelium and the neural crest (via *Wnt1-Cre*). Only conditional mutants generated using *Pax7^−Cre^* or *Wnt1-Cre* developed early onset congenital hydrocephalus due to stenosis of the third ventricle, suggesting that loss of neuroepithelial Pax3 is sufficient to disturb third ventricle morphogenesis. Dilation of lateral ventricles occurs as early as E14.5, and lineage-mapping revealed that the neuroepithelial cells in the conditional mutants are present, but fail to undergo normal differentiation at the stenotic site. Concomitant with a narrowing of the mutant third ventricle, we detected ectopic apoptosis, reduced proliferation, and abnormal β-catenin localization. Furthermore, consistent with the overlapping expression pattern of Pax3 and Pax7 in early cranial neuroepithelium, we demonstrated a combinatorial role, as compound *Pax3/Pax7* heterozygotes display partially-penetrant congenital hydrocephalus. These murine data provide an experimental paradigm underpinning clinical observations of the presence of PAX3 mutations in some hydrocephalic patients.

## 1. Introduction

Pax3 is a transcription factor expressed in the neural tube, neural crest (NC), and somites during early embryogenesis. The importance of Pax3 is well established by the *in utero* and neonatal death of Pax3 homozygous mutants [1,2] or hypomorphs [3] with an 80% reduction of Pax3 expression. However, this early death and the associated composite structural defects hinders our understanding of the functional role Pax3 plays in any given tissue/organ, especially subsequent defects which might not manifest until postnatal life [1,2,3,4].

The NC lineage distributes derivatives to a diverse range of organs and tissues, including the brain and cranial structures. In *Pax3*-deficient mice NC deficiency, and subsequent lack of colonization, results in lost or diminished tissues [1,5]. In the head, deficiency of *Pax3* leads to failed neural tube closure, resulting in exencephally which disrupts cranial development. In addition to the connective tissue and cranium, the NC also contributes to the meninges and pericytes of some cranial vasculature [6]. The dysmorphic cranial structure in systemic *Pax3* nulls makes it impossible to study cranial structural and/or functional defects in postnatal life. In order to bypass the well-known cardiovascular-associated mid-gestation lethality of homozygous *Pax3* nulls [3,5] and to determine the function of Pax3 in postnatal neural development, we crossed mice carrying a *Pax3^flox/flox^* conditional allele with *Wnt1-Cre* mice [7]. Embryonic Cre-mediated recombination removes *Pax3 exon5*, which is flanked by loxP sites. This deletion results in a Pax3 protein that is predicted to be non-functional due to introduction of a premature stop codon [8]. *Wnt1-Cre* is extensively used to study development of the NC and its derivatives, as well as subsequent brain and craniofacial growth, as *Wnt1-Cre* expression is restricted to the dorsal neuroepithelium [7]. Significantly, although *Wnt1-Cre* is robustly expressed in the dorsal neural tube prior to NC emigration, our previous studies revealed that *Wnt1-Cre* mediated deletion of *Pax3* did not cause *in utero* lethality or affect cardiac, craniofacial, or dorsal root ganglia development, which are dependent upon NC colonization [9]. However, we did find that despite haploinsufficient conditional *Pax3^Δ5/f^/Wnt1-Cre* mutants being present at normal Mendelian ratios at birth, roughly half of these mutants exhibit exencephaly and die perinatally [9]. Unexpectedly, we also observed that the remaining *Pax3^Δ5/f^/Wnt1-Cre* neonates that did not exhibit a cranial neural tube closure defect developed early-onset hydrocephalus.

Hydrocephalus, an accumulation of cerebrospinal fluid in the cranial cavity due to obstruction of the flow in and out of the cavity, is one of the most common birth defects. Thus, in order to generate conditional *Pax3* mutants in a wild-type environment, *Pax3^flox/flox^* mice were crossed with *Wnt1-Cre*, but without a *Pax3^Δ5/+^* allele. Significantly, all *Pax3^flox/flox^*/*Wnt1-Cre* mutants exhibited an early hydrocephalus phenotype. The postnatal viability of these genetically-defined mutants enabled us to identify the primary pathology as a disruption in the homeostasis of cerebrospinal fluid. Additionally, conditional *Pax3* targeting using *Pax7^−Cre^* [10] and *P0-Cre* [11], which separately mark only neuroepithelium or NC, respectively, enabled us to define the neuroepithelium as the primary site where the loss of Pax3 causes hydrocephalus. Of clinical significance, we show that simultaneous heterozygous mutation of *Pax7*, a *Pax3* paralog, induces hydrocephalus in compound heterozygous *Pax3/Pax7* mice. Our study thus provides the first experimental data to understand the etiology of hydrocephalus in some patients with known PAX3 mutations [12,13].

## 2. Experimental Section

### 2.1. Mice

*Pax3-*floxed [8] mice were intercrossed with *Wnt1-Cre* transgenic [7], *P0-Cre* transgenic [11], and *Pax7^−Cre^* knock-in mice [10] to generate lineage-restricted conditional mutants. Resultant offspring were PCR genotyped and analyzed, as described previously [3,9,14]. For lineage-mapping and to assess Cre-mediated recombination efficiency, *Pax3-*floxed mice were bred onto a homozygous *R26r* indicator background [15]. Both *Pax3* [8] and *Pax7* [14] floxed mice were crossed with germline Cre mice to obtain *Pax3^+/Δ5^* and *Pax7^+/Δ2^* heterozygous offspring that were subsequently crossed to generate compound heterozygous mutants. *Pax7^+/Δ2^* heterozygotes were also intercrossed to generate viable homozygous *Pax7^Δ2/Δ2^* offspring. These mice are maintained on a mixed genetic background at the IUSM Animal Facility. All procedures performed were in accordance with IACUC approved protocols and rules.

### 2.2. Histology, Immunohistochemistry, X-gal Staining, and in Situ Hybridization

Isolation of tissues, 4% paraformaldehyde fixation, processing, X-gal staining, immunostaining for anti-β-galactosidase [16,17], paraffin embedding, and serial sectioning were performed as described [3,9]. Samples at embryonic day (E) 17.5 and older were decalcified in 0.5M EDTA prior to processing for paraffin embedding. Additionally, slices of fixed brains were analyzed for gross morphology. Routine hematoxylin and eosin staining and immunostaining using ABC kit (Vectorstain, Burlingame, CA, USA) with DAB and hydrogen peroxide as chromogens was performed as described [3]. Dilution of primary antibodies was 1:200 for goat anti-Pax3 (Santa Cruz sc-7748, Dallas, TX, USA); 1:200 for mouse anti-Pax7 (Hybridoma Bank, Iowa City, IA, USA); 1:25 for monoclonal rat anti-Ki67 (DAKO, Carpinteria, CA, USA); 1:1000 for rabbit anti-β-galactosidase (Molecular Probes, Eugene, OR, USA); and 1:200 for rabbit anti-β-Catenin (Sigma, St. Louis, MO, USA). As the goat anti-Pax3 antibody can recognize both Pax3 and Pax7, we stained *Pax7* nulls as a control to confirm the respective pattern of Pax3 in E10 embryos. Apoptotic cells were detected using a FragEL™ DNA Fragmentation Detection Kit (Calbiochem, San Diego, CA, USA) as previously reported [3], and the epidermis was used as an internal control for DNA-break labeling efficiency. Radioactive S^35^-labeled *in situ* hybridization of *Pax3* (GenBank accession number NM_008781) was performed using an *exon5*-specific probe (to verify the absence of floxed transcript) and a *Pax3* full length probe (that detects both *exon5-*deleted mutant and wild-type transcripts) as previously described [9]. A corresponding sense probe was used as a control for specificity of the analysis. For each assay, whole embryos, neonates, and/or serial sections were examined in at least three individuals of each genotype at each stage of *in utero* development and postnatal morphogenesis. Wild-type and Cre-only littermates were used as age-matched controls.

### 2.3. Skeletal Preparations

Mice were sacrificed by carbon dioxide asphyxiation, skin removed, and heads incubated in Alcian blue and Alcian red stains as previously described [18]. Heads were transferred to 1% potassium hydroxide to clear tissue, then placed in glycerol containing 1% potassium hydroxide and subsequently stored in 50% glycerol. For this assay, at least three individual Alcian red/blue stained heads of each genotype were examined.

### 2.4. Western Blot Analysis

For Western blot examination, individual E10.5 embryos (*n* = 3–5 of each genotype) were homogenized in a 300 µL protein lysis buffer as described [3,9]. For each sample, 50 µL was loaded and run on a 10% SDS-PAGE gel (Bio-Rad, Hercules, CA, USA), transferred to nitrocellulose, and probed with mouse monoclonal anti-Pax3 (1:2000 dilution) or monoclonal anti-Pax7 (1:2000 dilution) antibodies (both obtained from the Hybridoma Bank) in a blocking solution. The signal was detected via ECL^Plus^ (Amersham, Piscataway, NJ, USA) with peroxidase-conjugated goat anti-mouse secondary antibody (1:5000 dilution, Promega, Madison, WI, USA). To verify equal loading, all blots were subsequently stripped, washed, re-blocked, and then probed with mouse anti-alpha-Actin antibody (1:5000 dilution, Sigma). X-ray films were scanned and densitometric quantification of signal intensity measured using ImageJ software. Statistical analysis was performed with Prism software (V5.02, Graphpad Software, La Jolla, CA, USA).

## 3. Results

### 3.1. Wnt1-Cre Restricted Pax3 Deletion Causes Perinatal Hydrocephalus

**Figure 1:**
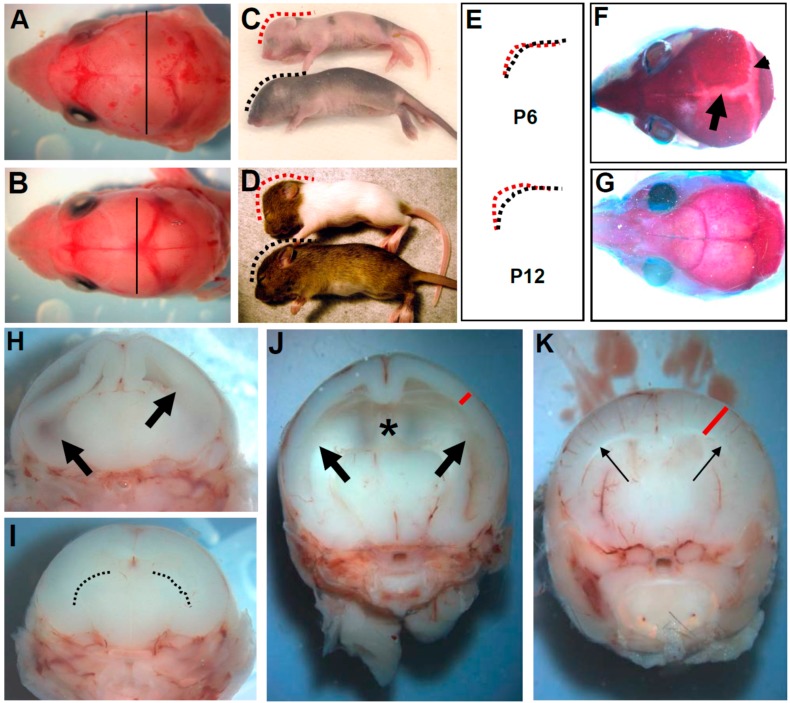
*Pax3^flox/flox^/Wnt1-Cre* conditional knockout mice exhibit congenital hydrocephalus, indicating that restricted loss of Pax3 within both the neural tube and migratory neural crest is sufficient to cause defects. (**A**,**B**) Dorsal view of postnatal (P) day 1 heads in *Pax3^flox/flox^/Wnt1-Cre* (**A**) and control (**B**) littermates. Lines drawn at similar anatomical planes measure the width of the heads, showing mild enlargement in mutants (**A**); (**C**,**D**) Lateral view of P6 (**C**) and P12 (**D**) *Pax3^flox/flox^/Wnt1-Cre* (top) and control (bottom) littermates. Note the generalized pigmentation defect in mutants; (**E**) Dashed lines from **C**,**D** illustrate the domed (red) mutant cranium; (**F**,**G**) Dorsal view of skeletal preparation of P20 *Pax3^flox/flox^/Wnt1-Cre* (**F**) and control (**G**) heads; Note sagittal (arrowhead) and transverse (arrow) suture fusion defects in only the mutants (**F**); (**H**,**I**) Transverse views of P1 heads from animals in (**A**,**B**); significant dilation of lateral ventricle (arrows) is already present in *Pax3^flox/flox^/Wnt1-Cre* mutants (**H**); dashed lines in control (**I**) indicate normal lateral ventricles; (**J**,**K**) Transverse view of P6 heads. Further dilation of lateral ventricles (large arrows) and loss of interventricular tissue (asterisk) is present in *Pax3^flox/flox^/Wnt1-Cre* mutants (**J**). As a consequence of expansion of mutant lateral ventricle, a thinned cortex (red line) also results.

Phenotyping revealed 100% (*n* = 50/50) of *Pax3^flox/flox^*/*Wnt1-Cre* mutants exhibit an early hydrocephalus phenotype (Figure 1, Table 1). *Pax3^flox/flox^*/*Wnt1-Cre* mice were born at the expected Mendelian ratio, but all died within 30 days of birth (*n* = 50/50). Soon after birth, P1 *Pax3^flox/flox^*/*Wnt1-Cre* pups displayed mildly enlarged heads, which became domed by P6 onwards, a sign of hydrocephalus (Figure 1). As cranial enlargement progressed, the mutant heads became more disproportional to their body size. *Pax3* conditional knockout brains accumulated cerebrospinal fluid in the lateral ventricles, which often collapsed during brain isolation (Figure 1H). Analysis of gross mutant brain morphologies indicated enlarged hemispheres, abnormal sutures, and a thinned cortex (Figure 1H,J) relative to aged-matched control littermates. Similar to other reported mouse models with hydrocephalus [19,20,21], the mutants displayed a reduction in growth evident as early as P6 (Figure 1C) and progressive wasting was accompanied by ruffled coats and dehydration from P12 onwards (Figure 1D). The ratio and phenotype of *Pax3^flox/flox^*/*Wnt1-Cre* mutants was the same regardless of whether *Cre* was carried by the father or mother. However, the other three genotypes (*Pax3^flox/+^*/*Wnt1-Cre*, *Pax3^+/+^*/*Wnt1-Cre*, and *Pax3^flox/flox^*) are unaffected and fertile and lived a normal life-span. As expected, and as previously shown [9], the *exon5*-specific *Pax3* probe did not detect a signal in the dorsal-most region of the neural tube in E10.5 and E14.5 *Pax3^flox/flox^*/*Wnt1-Cre* mutants that corresponded to the known *Wnt1-Cre*-expression domain (data not shown). Moreover, *Pax3^flox/flox^*/*Wnt1-Cre* pups exhibited a generalized pigmentation defect (Figure 1D), comparable to that reported in haploinsufficient conditional *Pax3^Δ5/f^/Wnt1-Cre* mutants [9], underscoring the efficient *Wnt1-Cre*-mediated recombination of the *Pax3-*floxed allele.

**Table 1:** Hydrocephalic incidence in mouse lines with conditional, systemic, or haploinsufficient mutations of *Pax3* and/or *Pax7*. Summary of hydrocephalus in the different mouse lines is indicated (*); the occurrence is sex-independent on mixed genetic background; presence of hydrocephalus (**) was determined by gross and/or anatomical (coronal sectioning) examination of postnatal animals one week old and beyond.

Mutant Line *	%Hydrocephalus **
*Wnt1-Cre:Pax3^flox/flox^*	100 (50/50)
*P0-Cre:Pax3^flox/flox^*	0 (0/20)
*Pax7^−Cre^:Pax3^flox/flox^*	100 (20/20)
*Pax3^+/**Δ5**^:Pax7^+/**Δ2**^*	40 (20/50)
*Pax3^+/**Δ5**^:Pax7^+/+^*	0 (0/100)
*Pax3^+/+^:Pax7^+/**Δ2**^*	0 (0/100)
*Pax3^+/+^:Pax7**^Δ2^**^/**Δ2**^*	0 (0/30)

### 3.2. In Utero Tissue Loss and Progressive Ventricular Dilation in Hydrocephalic Wnt1-Cre-Mediated Pax3 Mutants

**Figure 2:**
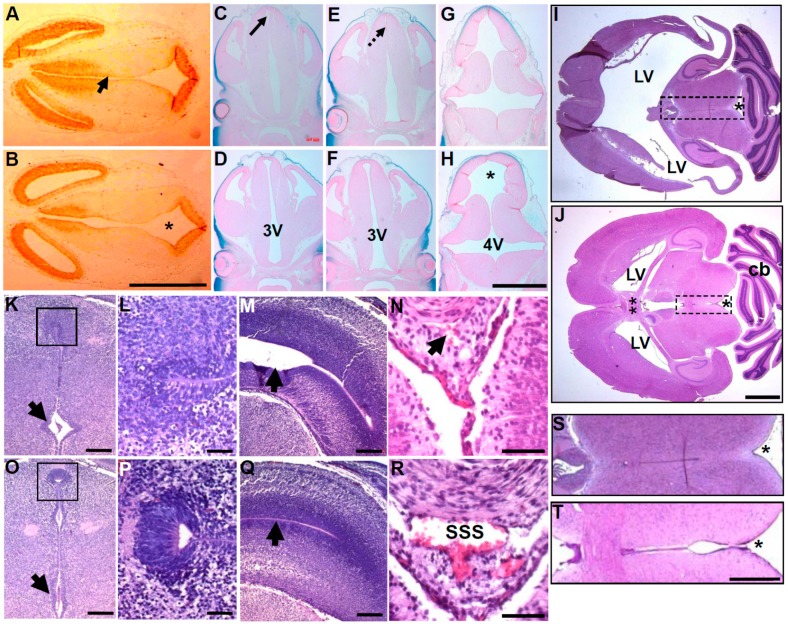
Histological analysis of ventricular dilation and tissue loss in *Pax3^flox/flox^/Wnt1-Cre* mutants. (**A**,**B**) Histology revealed a narrow third ventricle (arrow) along the E12.5 mutant (**A**) anterior-posterior axis compared to control (**B**); (**C**–**H**) E13.5 coronal sections from anterior to posterior and stained for *lacZ* reporter (blue) and eosin (pink) of *Pax3^flox/flox^/Wnt1-Cre;R26r* mutants (**C**,**E**,**G**) and *Wnt1-Cre/R26r* controls (**D**,**F**,**H**); Opening of the dorsal part of mutant third ventricle (3v) is narrow (arrow, **C**) or contracted (broken arrow, **E**) compared with the control (**D**,**F**). However, at this stage there is a connection between both mutant and control third ventricle and aqueduct (asterisk); (**I**,**J**) hematoxylin and eosin horizontal sections from P20 mutant (**I**) and control (**J**). Note mutant lateral ventricles (LV) are enlarged and fused due to loss of normal interventricular tissue (double asterisk, J); (**K**–**R**) hematoxylin and eosin stained transverse sections of E16.5 *Pax3^flox/flox^/Wnt1-Cre* (**K**–**N**) and control brains (**O**–**R**); **L**,**P** are magnified in areas indicated in **K**,**O** and clearly show diminished opening of dorsal part of the mutant third ventricle and an under-developed subcommissural organ (**K**,**L**); as a consequence of likely impaired fluid flow, the mutant medial region (arrow, **K**) is remarkably enlarged compared to control (arrow, **O**). Additionally, mutant lateral ventricle (arrow, **M**) is significantly dilated. Although the superior sagittal sinus (SSS) is easily recognizable in control (**R**) it is obscure and undersized in the *Pax3^flox/flox^/Wnt1-Cre* mutants (arrow, **N**); (**S,T**) enlarged images of boxed areas in **I**,**J** illustrate the lack of connection between the mutant third ventricles and aqueducts (asterisks) in mutants (**S**) but not in controls (**T**). Scale bars: A–D,F–I = 1mm; E,J = 2mm; S,T = 0.5mm.

Histology confirmed mutant (*n* = 10/10) lateral ventricles were massively dilated and the cerebral cortex was remarkably thin (Figure 2I). Moreover, there is a significant reduction in the mutant corpus callosum. However, the hippocampus, all four choroid plexuses, and the cerebellum (despite being largely derived from *Wnt1-Cre* expressing lineage [20]) are grossly similar between control and mutants from embryonic to postnatal stages (Figure 2). The fact that we did not find any defects in the *Wnt1-Cre*-positive cerebellum is reminiscent of our previous study that demonstrated that different Pax3-related tissues have a differential threshold sensitivity to alterations in Pax3 expression levels [3]. Significantly, a small superior sagittal sinus (which allows blood to drain from the lateral aspects of anterior cerebral hemispheres), an under-developed subcommissural organ, a narrowed E13.5 and diminished E16.5 opening between the third ventricle was observed in the conditional mutants. By P20, there was stenosis between the mutant third ventricle and aqueducts (Figure 2I), suggesting a progressive reduction of cerebrospinal fluid flow, both *in utero* and post-natally. Overproduction, impaired flow, or impaired absorption of cerebrospinal fluid, are all sufficient to cause hydrocephalus by creating a build-up of cerebrospinal fluid in the cerebral ventricular system. As we know, cerebrospinal fluid is most likely blocked at the stenotic site of the third ventricle upstream of diminished superior sagittal sinus. Although the superior sagittal sinus collects cerebrospinal fluid prior to entering the blood stream and has been shown to directly cause hydrocephalus in mice [22], it is unlikely that a defective superior sagittal sinus is causing the observed cerebrospinal fluid build-up in *Pax3^flox/flox^*/*Wnt1-Cre* lateral ventricles. These histological analyses established the prenatal onset of third ventricular stenosis as a primary cause of congenital hydrocephalus in these mutants. Lack of subcommissural organ or abnormal subcommissural organ morphogenesis is a common feature in mice that display congenital hydrocephalus [20,23]. Combined, these data indicate that the hydrocephalus phenotype of *Pax3^flox/flox^*/*Wnt1-Cre* mice was caused by defects within the function and structure of the cerebrospinal fluid drainage system beginning *in utero*.

### 3.3. Wnt1-Cre Lineage Mapping Reveal that Ventricular Neuroepithelium and Subcommissural Organ Morphogenesis Requires Pax3

**Figure 3:**
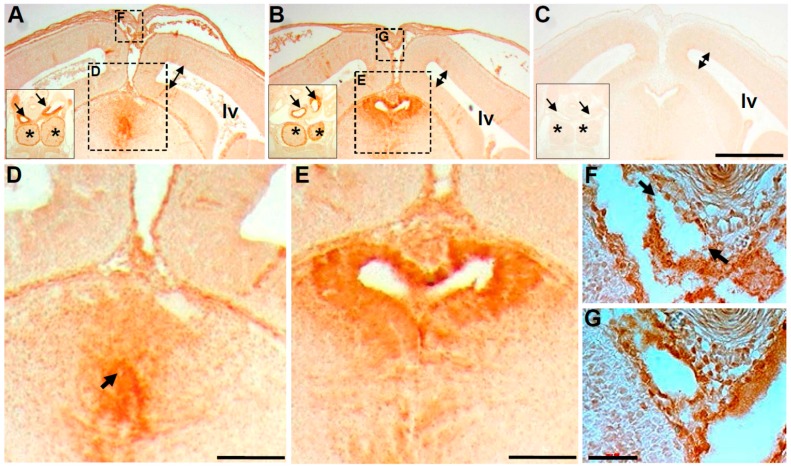
Lineage mapping reveals restricted loss of *Pax3* results in abnormal opening to the brain third ventricle. (**A**–**E**) Coronal sections of E14.5 of *Pax3^flox/flox^/Wnt1-Cre; R26r* mutant (**A**,**D**), *Wnt1-Cre/R26r* control (**B**,**E**) and *Pax3^flox/flox^/R26r* control (**C**) littermates following β-galactosidase (brown) immunohistochemistry to unequivocally detect *lacZ* expression. The *Wnt1-Cre*-negative sample (**C**) serves as a negative control. Significantly, the epithelium lining the control third ventricle and subcommissural organ is specifically labeled (**B**), whereas in the mutant (**A**), although a group of cells is positively labeled, they failed to form a normal tubular structure in the mutant. Enlarged views from **A**,**B** (indicated via large dotted line boxed areas labeled **D**,**E**) reveal *lacZ*-expressing elongated columnar epithelial cells surrounding a clear opening in control (**E**) but *lacZ*-expressing mutant cells fail to establish the normal opening to the third ventricle (arrow in **D**). Additionally, double-headed arrows indicate the space in the lateral ventricles in all three genotypes, revealing a mild dilation of lateral ventricles in only the mutant (**A**). Inserts in **A**,**B** (solid line boxes) show a similar pattern of typical *Wnt1-Cre/R26r* expressing cells in both mutant and control great arteries (arrows) and thymus (asterisk), from more posterior sections adjacent to the outflow tract of the heart. (**F**,**G**) High-power view from small dotted line boxed areas in **A**,**B**, demonstrating that the superior sagittal sinus (SSS) and surrounding tissue are positively labeled for *Wnt1-Cre/R26r* lineage. Note however that the circumferential labeling pattern seen in control (**G**) is discontinuous in the mutant (arrows, **F**). Scale bars: A–C = 400 µm; D,E = 130 µm; F,G = 50 µm.

*Wnt1-Cre* mediated *Cre* expression is known to be expressed in both the dorsal neuroepithelium before NC delamination and within migratory NC and their derivatives [7]. Thus, to determine whether the observed stenosis of third ventricle is directly related to previous *Wnt1-Cre*-mediated *Pax3* deletion during early embryonic cranial development, we mapped expression of β-galactosidase, the *R26r* reporter designed to trace cells derived from *Wnt1-Cre*-expressing lineage [15]. Rather than using the conventional whole mount assay, which raises concerns of substrate penetration and tissue integrity associated with sample preparation and fixation artifacts, we detected the reporter via immunohistochemistry in serial paraffin sections. As expected, due to unaffected cardiovascular histology and survival at birth, smooth muscle cells around the great arteries and the epithelium around the *Pax3^flox/flox^*/*Wnt1-Cre* thymic cortex were positively labeled (Figure 3A insert). Although E14.5 control brain elongated epithelial cells lining the opening of dorsal third ventricle and subcommissural organ are all positively labeled (Figure 3B,E), Pax3 conditional mutants do not exhibit corresponding expression patterns (Figure 3A,D). Despite there being β-galactosidase-positive cells, they are not elongated and fail to form a normal epithelial opening, particularly in the posterior segment of the third ventricle (as the mutant anterior segment appears less affected). Concomitant with the observation of an obstructive third ventricle, there is mild dilation of the mutant lateral ventricles at this developmental stage, further supporting the suggestion that discontinuous third ventricular obstruction is the primary cause of hydrocephalus. Cells surrounding the superior sagittal sinus are also positively labeled in the control (Figure 3G) and, although the mutant superior sagittal sinus does not show a diminished opening at E14.5, it is evident that β-galactosidase labeling is discontinuous. The mutant superior sagittal sinus lumen is also irregularly shaped. Together, these results indicate that stenosis of the posterior segment of the third ventricle and attendant aqueduct defects, along with secondary abnormalities in the superior sagittal sinus, underlie the congenital hydrocephalus in *Pax3^flox/flox^*/*Wnt1-Cre* mutants.

### 3.4. Molecular Marker Analysis Reveals Neuroepithelial Dysmorphia

**Figure 4:**
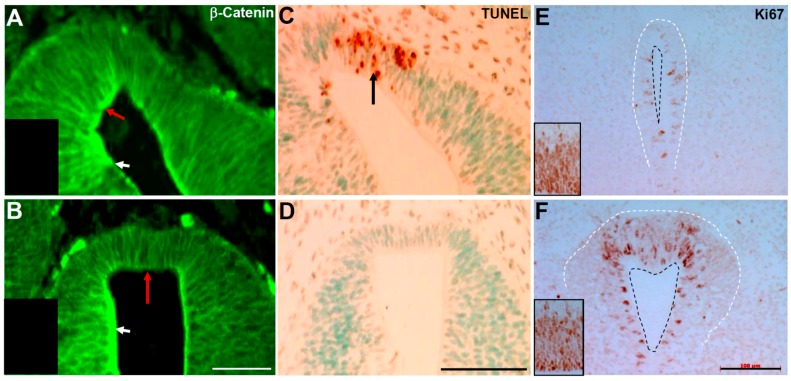
Marker analysis of anterior third ventricle morphogenesis. (**A**,**B**) Immunofluorescence detection of β-catenin expression in *Pax3^flox/flox^/Wnt1-Cre* mutants (**A**) compared to control (**B**) E12.5 embryos. Red arrows point to the apical pole of the anterior side of third ventricles, revealing robust β-catenin in mutants but not control. However, β-Catenin expression is equally present in control and mutant lateral walls (white arrows). Inserts are negative controls, illustrating lack of autofluorescence; (**C**,**D**) TUNEL assay shows ectopic apoptosis in anterior wall of third ventricle in *Pax3^flox/flox^/Wnt1-Cre* mutants (arrow, **C**) but none in control; (**E**,**F**) Ki67 immunohistochemical analysis of reduced cell proliferation in *Pax3^flox/flox^/Wnt1-Cre* mutants (**E**) compared to control (**F**) E12.5 embryos. Black dashed lines indicate the ventricular lumen and the white lines indicate the pseudostratified epithelium. Inserts from E12.5 lateral ventricles, exhibiting similar Ki67 labeling index in *Pax3^flox/flox^/Wnt1-Cre* mutants (**E**) and control (**F**). Scale bars: A,B = 50 µm; C–F = 100 µm.

As canonical Wnt/β-catenin signaling is known to establish polarity of epithelial cells and its structural absence [24] and conditional deletion [25] have been reported as primary causes of cell polarity loss resulting in hydrocephalus, we examined β-catenin localization (Figure 4A,B). Immunofluorescence revealed similar expression patterns in lateral and apical epithelial cells lining the E12.5 third ventricle along the anterior-posterior axis in both *Pax3^flox/flox^*/*Wnt1-Cre* and controls. However, the anterior wall of the mutant third ventricle had ectopic up-regulated β-catenin on its apical side, when compared to controls (Figure 4A,B). Significantly, ectopic β-catenin localization in the spinal cord can alter normal neuroepithelial patterning and function [26]. Moreover, *β-catenin* *^flox/flox^/Pax3-Cre* mutants exhibit caudal neural tube closure defects and transgenic activation of *Pax3* cDNA can rescue these neural tube defects in *β-catenin* conditional mutants [27]. Combined with our mis-expression results, these data suggest that precise expression of both β-catenin and Pax3 is needed for normal cranial and caudal neuroepithelial morphogenesis.

Given the lack of a detectable subcommissural organ and stenosis of the posterior segment of the third ventricle and aqueduct (see Figure 2 and Figure 3), we surveyed both neuroepithelial cell death and proliferation. TUNEL revealed ectopic apoptosis in the anterior wall of the third ventricle in *Pax3^flox/flox^*/*Wnt1-Cre* mutants (Figure 4C) that was not present in control littermates (Figure 4D). Conversely, immunolabeling with Ki67 (Figure 4E,F), a cell proliferation marker, revealed a significant reduction of cell proliferation in the third ventricle neuroepithelium around the diminished lumen opening in *Pax3^flox/flox^*/*Wnt1-Cre* mutants (Figure 4E). Given a well-established association of *Pax3* reduction and apoptosis in the neural tube [28] along with initial suppression of neuroepithelial proliferation in systemic *Pax3* nulls exhibiting neural tube closure defects [29], we might hypothesize that *Pax3*-deficent aberrant proliferation/apoptosis is a fundamental cellular mechanism instigating hydrocephalic pathogenesis. Collectively, published data and our marker analysis suggest Pax3 may play a role in specifying third ventricle neuroepithelial morphogenetic processes and that lack of *Pax3* results in ectopic β-catenin expression, cell death, and uncontrolled epithelial cell proliferation.

### 3.5. Restricted Loss of Pax3 within Migratory Neural Crest Does not Cause Hydrocephalus

**Figure 5:**
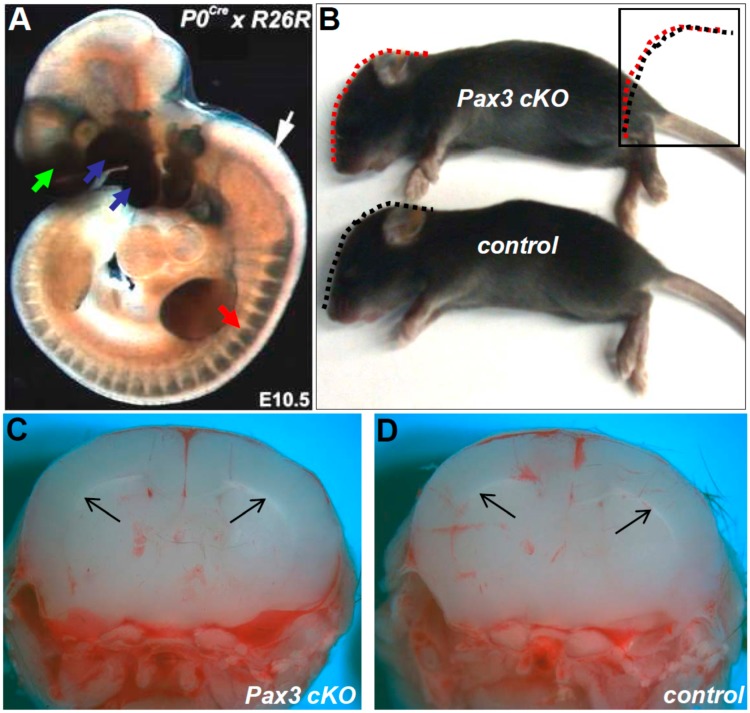
*Pax3^flox/flox^/P0-Cre* conditional knockout mice do not exhibit congenital hydrocephalus, indicating that restricted loss of Pax3 within the migratory neural crest is insufficient to cause defects. (**A**) *P0-Cre/R26r* lineage mapping in E10.5 control embryos reveals that the neural crest-colonized tissues, such as cranial-facial (green arrow), pharyngeal arches (blue arrows), and dorsal root ganglia (red arrow) are all positively labeled (blue staining). However, as *P0-Cre* is only expressed in neural crest derivatives after emigration from the neural tube, *P0-Cre/R26r* lineage mapping verified that the neural tube (white arrow), hindbrain, and brain are not labeled; (**B**) lateral view of P12 *Pax3^flox/flox^/P0-Cre* (upper) and control (lower) littermates. Their lateral cranial profile is indistinguishable (indicated by dotted lines and compared side-by-side in insert). Also note normal pigmentation of *Pax3^flox/flox^/P0-Cre* neonate: (**C**,**D**) coronal views of neonates in **B**, verifying *Pax3^flox/flox^/P0-Cre* neonate lateral ventricles (arrows in C) are unaffected.

Given that *Wnt1-Cre* simultaneously marks both neuroepithelium and NC, and defects related to their derived structures are present in *Pax3^flox/flox^*/*Wnt1-Cre* mutants, we sought to limit Pax3 deletion to the migratory NC lineage only. Thus, we used *P0-Cre* mice that express Cre in migrating NC only from E9.0 onwards [11] to test the effects of removing Pax3 within NC only, once they have emigrated from the neuroepithelium. Conditional *Pax3^flox/flox^*/*P0-Cre* mice were born at the expected Mendelian ratio, do not exhibit any differences in lateral ventricles, and are hydrocephalus-free (*n* = 0/20) throughout their normal life span (Figure 5, Table 1). Thus, the impact of Pax3 mutation upon hydrocephalus pathogenesis lies in the neuroepithelium which gives rise to the third ventricle and not the *P0-Cre*-marked NC lineages.

### 3.6. Neuroepithelial Deletion of Pax3 is Sufficient to Cause Hydrocephalus

**Figure 6:**
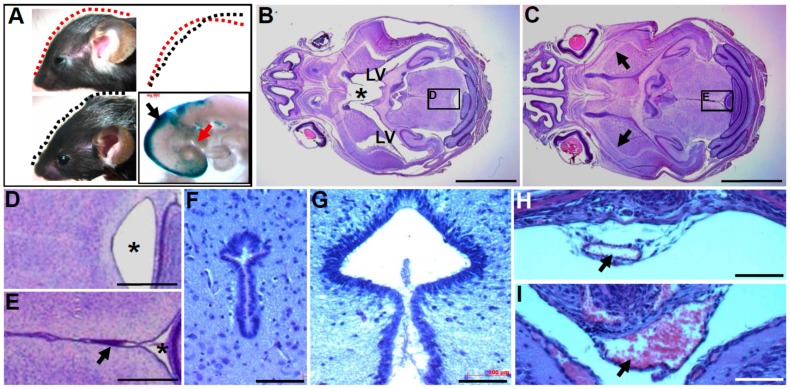
*Pax3^flox/flox^/Pax7^−Cre^* conditional knockout mice exhibit congenital hydrocephalus, indicating that restricted absence of Pax3 from the *Pax7* lineage is sufficient to cause defects. (**A**) Lateral views of *Pax3^flox/flox^/Pax7^−Cre^* (top) and control (bottom) P20 pups, with lateral profiles depicted by dotted lines and compared side by side to highlight the domed cranium in *Pax3^flox/flox^/Pax7^−Cre^* mutant. Insert depicts *Pax7^−Cre^*/*R26r* lineage mapping in an E9.5 control embryo, with positive *lacZ-*labeling (blue) in the cranial neural tube (black arrow) with negligible labeling of neural crest structures (red arrow); (**B**–**I**) Hematoxylin and eosin sections of P6 *Pax3^flox/flox^/Pax7^−Cre^* (**B**,**D**,**F**,**H**) and control (**C**,**E**,**G**,**I**) littermates. Note that mutant lateral ventricle is dilated and loss of tissue is apparent (**B**). Enlarged views of boxed areas in (**B**,**C**) reveals the lack of connection between the mutant third ventricle and aqueduct (asterisk, **D**) compared to control littermate (**E**); Arrow in E indicates the control posterior part of the third ventricle connecting to the aqueduct (asterisk). When coronal sections of the posterior part of the control (**G**) third ventricles are compared to *Pax3^flox/flox^/Pax7^−Cre^* mutants (**F**), the opening of the mutant ventricle is significantly narrowed; high-power views of the mutant (**H**) and control (**I**) superior sagittal sinus (arrows) confirm that the mutant lumen size is significantly reduced. Scale bars: B,C = 2 mm; D,E = 500 μm; F–I = 100 μm.

As the above results using *Wnt1-Cre* and *P0-Cre* suggest hydrocephalus likely results from Pax3 mutation within the neuroepithelium, we sought to experimentally test this hypothesis. Using *Pax7^−Cre^* knock-in line [10], we generated *Pax3^flox/flox^*/*Pax7^−Cre^* mutants (Figure 6). *Pax3* and *Pax7* are the most structurally and functionally related Pax members [4] and are expressed in the embryonic nervous system and somites. Both are co-expressed in the early dorsal neural tube [30,31] and share expression domains in the fetal brain and spinal cord. However, whereas *Pax3*-null fetuses exhibit exencephaly [1,5], *Pax7*-null mice survive to adulthood and the *Pax7-*null adult cranium and CNS are unaffected [14]. Significantly, the *Pax7^−Cre^* allele was generated by placing *Cre* cDNA at the 3’ UTR of the *Pax7* gene to enable normal expression of wildtype Pax7 protein in *Pax7^−Cre^* offspring. Moreover, *Pax7^−Cre^* is only expressed within the neuroepithelium and somites [10]. Meaningfully, 100% (*n* = 20/20) of the *Pax3^flox/flox^*/*Pax7^−Cre^* mutants exhibit early hydrocephalus (Figure 6, Table 1).

Lineage mapping confirmed that *Pax7^−Cre^*/*R26r* indeed marks neuroepithelium, but not NC, during early cranial development (Figure 6A). *Pax3^flox/flox^*/*Pax7^−Cre^* mutants are born at the expected Mendelian ratio, exhibit a fully-penetrant cranial phenotype, and die within a similar time window as *Pax3^flox/flox^/Wnt1-Cre* mutants. Although Pax3 and *Pax7^−Cre^* are both present in muscle cells, the lack of mutant musculature phenotypes may be due to the fact that *Pax7^−Cre^* is only expressed after endogenous Pax3, and that *Pax7^−Cre^* is only present in a subpopulation of Pax3-expressing muscles [10,14]. In order to verify Pax7 expression of the modified *Pax7* allele, we performed Western blot analysis at E10.5 to compare *Pax7^Cre/Cre^* vs *Pax7^+/+^* and, as expected [10], we confirmed similar levels of Pax7 protein when compared to wild-type. Histological analysis confirmed a similar obstructive defect in the posterior part of mutant third ventricles (Figure 6B,D,F), dilated lateral ventricles, as well as tissue loss and reduced superior sagittal sinus lumen size (Figure 6H). Interestingly, although *Pax7^−Cre^*/*R26r* lineage-mapping does not show *Pax7^−Cre^* derivatives in the normal superior sagittal sinus or surrounding tissues, *Pax3^flox/flox^*/*Pax7^−Cre^* mutants exhibit diminished superior sagittal sinus (Figure 6H). Thus, defective superior sagittal sinus morphogenesis is likely to be a secondary consequence in both *Pax3^flox/flox^/Wnt1-Cre* and *Pax3^flox/flox^*/*Pax7^−Cre^* mutants.

### 3.7. Pax3 May Play a Role in Regulating Cell Proliferation within the Epithelium

**Figure 7:**
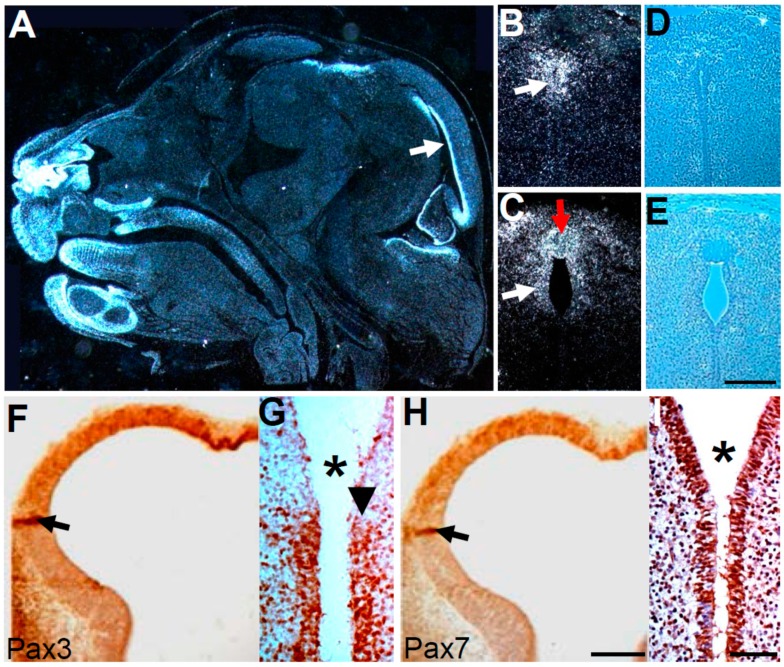
Analysis of Pax3 and Pax7 spatiotemporal expression patterns, Cre expression limits and cell proliferation during cerebral ventricular development. (**A**–**C**) *In situ* hybridization detection of *Pax3* mRNA in E15.5 wild-type heads show active expression in ventricular epithelium (arrow) and multiple craniofacial structures (**A**). Coronal E14.5 sections cut at the subcommissural organ (SCO, indicated by the red arrow in **C**) level of *Pax3^flox/flox^/Wnt1-Cre* mutant (**B**) and control (**C**) reveal *Pax3* is still expressed in the ventricular epithelium (white arrow) comparable to control (**C**), despite collapse of the ventricular opening in mutants; (**D**,**E**) Phase contrast images of **B**,**C**, illustrating the lack of a distinct subcommissural organ in the mutant **D**); (**F**,**H**) Immunohistochemical detection of Pax3 (**F**) and Pax7 (**H**) protein on adjacent telencephalic vesicle sections at E10, reveals co-localization in the majority of the neuroepithelium, with especially robust expression (brown) in the lamina terminalis. The black arrows point to intense artificial staining due to tissue folding; (**G**,**I**) Immunohistochemical detection beta-galactosidase expression following lineage mapping at E13.5 for *Pax7^−Cre^/R26r* (**G**) and *Wnt1-Cre/R26r* (**I**) following Cre-mediated activation of *R26r* reporter. While *Wnt1-Cre* marks the ependymal layer from third ventricle (3v) through aqueduct (**I**), *Pax7^−Cre^* only marks the ependymal layer of 3v (arrow points to the 3v-aqueduct junction and asterisk indicates aqueduct). Scale bars: B–E = 100 µm; F,H = 200 µm; G,I = 50 µm.

Pax3 expression mostly abates upon cell differentiation [32] and is generally thought of as an early embryonically-expressed transcription factor [1,5]. Thus, we used radioactive *in situ* hybridization analysis to spatiotemporally map *Pax3* expression in embryonic and fetal brains. Notably, we found that control ventricular epithelium still expresses high levels of *Pax3* mRNA at E15.5 (Figure 7A), in addition to the known early dorsal neural tube expression from E8.0 onwards [9]. This implicates a continued fetal role for Pax3 in cranial morphogenetic processes at late developmental stages. *In situ* hybridization with full length *Pax3* cRNA, which labels both mutant and wild-type mRNA, detects similar expression patterns in both control and *Pax3^flox/flox^/Wnt1-Cre* mutant posterior third ventricle neuroepithelium (Figure 7B,C), thus showing that *Pax3*-expressing epithelial cells are present in both mutants and controls at E14.5. However, a *Pax3*-positive subcommissural organ is barely detectable in the mutant (Figure 7B). Whether Pax3 is required for maintaining the third ventricle opening via regulation of cell proliferation, as suggested by reduced cell proliferation in the mutant posterior third ventricle (Figure 4), remains unknown. Further refined tissue-specific approaches and temporally-controlled deletion of *Pax3* will be required to define the spatiotemporal window as to when and where Pax3 is physiologically required during fetal brain maturation.

As *Pax3^flox/flox^*/*Pax7^−Cre^* mutants exhibit hydrocephalus similar to *Pax3^flox/flox^/Wnt1-Cre* mutants, we examined Pax3 and Pax7 proteins (Figure 7F,H). In E10 control telencephalic vesicle sections, there is co-localization in the majority of the neuroepithelium, with especially robust expression in the lamina terminalis. Given the anterior wall of the third ventricle develops from the lamina terminalis and that the lamina terminalis expresses higher levels of Pax3 than the flanking epithelia in early brain development (Figure 7F), our analyses suggest Pax3 plays an important role specifically in third ventricle anterior wall morphogenesis. Moreover, detailed *R26r* lineage-mapping revealed an overlapping distribution pattern of epithelial cells marked by both *Wnt1-Cre* and *Pax7−Cre* in the third ventricle neuroepithelium (Figure 7G,I), underscoring why similar cranial defects occur at the same site in both *Pax3^flox/flox^/Wnt1-Cre* and *Pax3^flox/flox^*/*Pax7^−Cre^* mutants.

### 3.8. Pax3 and Pax7 Play a Combinatorial Role during Third Ventricle Morphogenesis

**Figure 8:**
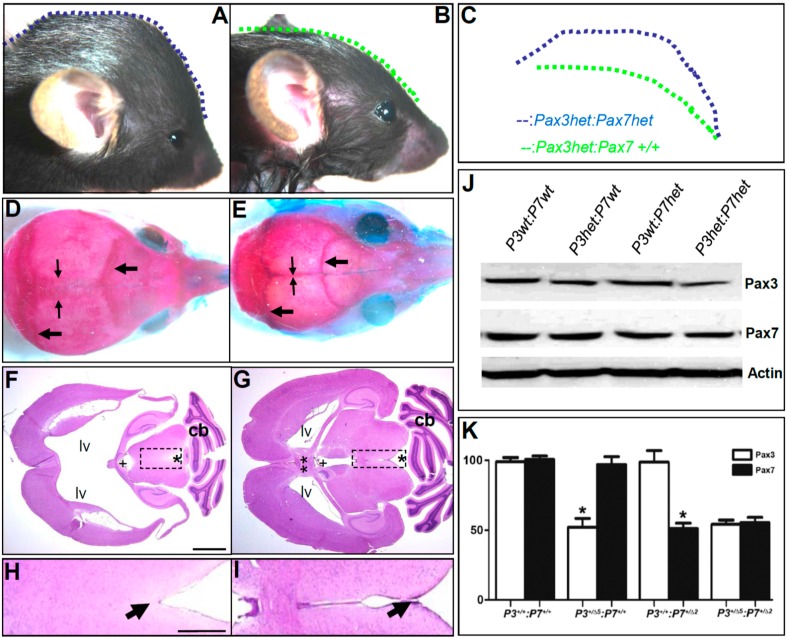
Pax3 and Pax7 play a combinatorial role in cerebral ventricular development. (**A**–**C**) Lateral views of *Pax3^+/Δ5^/Pax7^+/Δ2^* compound heterozygous mutant (**A**) and control (**B**) P20 littermates, with lateral profiles depicted by dotted lines and compared side-by-side to highlight the large domed cranium in *Pax3^+/−^/Pax7^+/−^* mutant (**C**); (**D**,**E**) Skeletal preps of heads from animals in (**A**,**B**); only the compound mutant (**D**) displays sagittal (narrow arrows) and transverse (thick arrows) suture fusion defects; (**F**,**G**) Hematoxylin and eosin sections reveal that the mutant lateral ventricle (**F**) is dilated and there is a loss of structure, when compared to control (double asterisks, G). The normal anterior segment of the third ventricle (plus sign), aqueduct (asterisk), and cerebellum (c,b) are indicated; (**H,I**) high power view of stenotic connection between mutant third ventricle and aqueduct (arrow, H), compared to normal third ventricle-aqueduct junction in control (**I**); (**J**,**K**) Results of representative Western blot analysis (**J**) and densitometric quantification (**K**) of Pax3 and Pax7 expression levels in individual E10.5 whole embryo lysates of designated genotypes (*n* = 3–5 of each genotype were analyzed). Note the gene dosage-dependent reduction (~50%) of corresponding protein level in various heterozygous compound offspring, relative to wild-type (*P3^+/+^/P7^+/+^*). Data are represented as mean ± SEM, * *p* < 0.05 by Student’s t-test (Pax3 *versus* Pax7 in each genotype). Scale bars: F,G = 2 mm; H,I = 0.5 mm.

Pax7 is an orthologue of Pax3, and Pax7 has been shown to be able to replace Pax3 in the dorsal neural tube, NC, and during somite development [4]. Although heterozygous mutations of either Pax3 or Pax7 do not cause hydrocephalus and, along with the close relationship and overlapping expression pattern of the two Pax members in early cranial neuroepithelium (Figure 7), we questioned whether loss of one copy of *Pax7* would result in hydrocephalus within *Pax3^+/Δ5^* heterozygotes. *Pax7^+/Δ2^* heterozygous (in which exon 2 is deleted, resulting in a null mutation [14]) were bred to *Pax3^+/Δ5^* heterozygotes to generate compound heterozygous mutants. Analysis of stages P1-P30 revealed approximately 40% hydrocephalic cases among compound *Pax3^+/Δ5^/Pax7^+/Δ2^* mutants (*n* = 20/50) and 0% cases (*n* = 0/100) in control littermates bearing either *Pax3^+/Δ5^* or *Pax7^+/Δ2^* mutations in isolation (Figure 8, Table 1). Pathogenesis of *Pax3^+/Δ5^/Pax7^+/Δ2^* mutant hydrocephalus (Figure 7A–I) is grossly and anatomically indistinguishable from that observed in *Pax3^flox/flox^*; Wnt1-Cre (Figure 1 and Figure 2) and *Pax3^flox/flox^*/*Pax7^−Cre^* mutants (Figure 6). Given the partial penetrance of hydrocephalus in the compound heterozygous mutants, we were unable to systemically investigate its *in utero* origin. Nevertheless, the same defect in the third ventricle and suture fusion occurs and strongly suggests that affected compound heterozygous mutants follow a similar hydrocephalic pathogenesis. The finding of suture defects in both *Wnt1-Cre*-mediated mutants and compound heterozygotes, suggests that aberrant cranial bone development may contribute to the eventual phenotype. To confirm that expression levels of both Pax3 and Pax7 would be reduced in the compound heterozygous mutants, we performed Western blot analysis and revealed a gene dosage-dependent ~50% reduction for each protein in single E10.5 heterozygous animals and for both proteins in compound heterozygotes (Figure 8J,K). Taking advantage of *Pax7^Δ2/Δ2^* postnatal viability, we also investigated the effect upon brain morphogenesis in *Pax7^Δ2/Δ2^* nulls. However, examination of coronal sections at P20 (*n* = 0/30) did not reveal any anomalies indicative of hydrocephalus (Table 1), suggesting that overlapping and earlier expressed Pax3 can functionally compensate for loss of Pax7 during third ventricle development.

## 4. Discussion

### 4.1. Pax Family Genes are Required in Utero for Neuroepithelial Morphogenesis

Although not all forms of hydrocephalus are birth defects, more genetically-modified mouse models are being generated to mimic human congenital hydrocephalus, a prevalent and devastating disease [33]. By examining *Pax3* conditional mutants, we established abnormal narrowing of the third ventricle as a novel mechanism that leads to early onset congenital hydrocephalus, caused by stagnation of cerebrospinal fluid in the ventricles. Additionally, superior sagittal sinus abnormalities, similar to that reported in hydrocephalic *Pdn/Pdn* mice [22] are also present. Moreover, via use of *Pax3/Pax7* compound heterozygous mutations, we provide the first experimental mouse data to link the combinatorial mutation of Pax3 and Pax7 to occurrences of congenital hydrocephalus.

### 4.2. Functional Effects of Pax3 Mutation in Congenital Hydrocephalus Pathogenesis

Both *Wnt1-Cre* and *Pax7^−Cre^* drivers are robustly expressed in the embryonic neuroepithelium and both structural and molecular defects are present *in utero* (Figure 2, Figure 3 and Figure 4), and, as Pax3 expression mostly abates upon cell differentiation [32], the results of our study suggest that it is the specific absence of Pax3 that is instrumental in causing these congenital hydrocephalus phenotypes. Moreover, the *P0-Cre* lack of phenotype (Figure 5) supports the concept that once *Pax3*-expressing NC precursors have initiated epithelial-mesenchymal transformation and begun migration, Pax3 is no longer required during cranial morphogenesis and third ventricle development. Interestingly, although both cranial and caudal neural tube closure defects are consistently observed in *Pax3^Δ5/Δ5^* mutants [2,3,9] and mutations in PAX3 are etiologically associated with syndromic neural tube defects [13], neither *Pax3^Δ5/flox^/Wnt1-Cre* [9] or *Pax3^flox/flox^/Wnt1-Cre* conditional mutants exhibit spina bifida (Figure 1). Consistent with *Wnt1-Cre* expression initiating ~E8.0 head-to-tail and posterior NT closure occurring prior to onset of *Wnt1-Cre* expression [9], these data do not shed any light on whether the cranial neural tube is more affected than the caudal region by the loss of Pax3.

Within our model system, Cre-mediated deletion of *Pax3 exon5* creates a premature stop codon and loss of Pax3 homeodomain and its downstream sequence [8]. Moreover, Western blot analysis has previously confirmed that the full length Pax3 protein is absent in the homozygous *exon 5* mutants [9] and immunohistochemistry has verified the loss of wild-type Pax3 protein in *Pax3^flox/flox^/ Wnt1-Cre* conditional mutants within the *Cre* expression domains [9,34]. This *Pax3* exon 5 allele does not result in any full length Pax3 expression, in which case, the congenital hydrocephalus phenotype is most likely due to lack of wild-type Pax3 within the dorsal neural tube. However, our data do not totally exclude the possibility of non-specific effects of a mutant Pax3 protein, as our Western blot data cannot necessarily confirm an absence of a truncated mutant protein, because the epitope that is detected by the monoclonal Pax3 antibody is predicted to be deleted in the mutant protein. Additionally, a mouse embryonic stem cell approach suggested that Pax3 fragments containing only the paired and octapeptide domains may still have some activity [35]. Nonetheless, our compound *Pax3/Pax7* heterozygote partial phenotypes support a loss-of-function role, as both are co-expressed in the neural tube and neither individual heterozygote mutant exhibits any gain-of-function defects. Further studies are required to determine what specific aspect of Pax3 and/or Pax7 orthologue function may be directly responsible for abnormal neuroepithelial morphogenesis, such as DNA-binding, trans-activation, p53 degradation and/or β-catenin signaling, which are all known to play direct roles in neural tube closure [10,27,28,29,31]. Collectively, published data and our analysis suggest Pax3 may play a direct role in specifying third ventricle neuroepithelial morphogenetic processes and that lack of Pax3 results in abnormal β-catenin expression, cell death, and uncontrolled epithelial cell proliferation.

Favoring the theory that hydrocephalus is an outcome of multifactorial risks, only ~40% of compound *Pax3^+/Δ5^*/*Pax7^+/Δ2^* mutants develop hydrocephalus (Table 1). This partial penetrance would, thus, make these compound mutants a biological sensor for screening genetic and non-genetic factors, exposure to which would predispose one to congenital hydrocephalus. As for environmental impact, human risk factors include a lack of prenatal care, multiparous gestation, maternal diabetes, maternal chronic hypertension, maternal hypertension during gestation, and alcohol use during pregnancy [36]. Moreover, familial patterns of hydrocephalus prevalence suggest that there are genes that increase risk [36]. Significantly, maternal diabetes has already been shown to reduce fetal *Pax3* mRNA expression through oxidative-stress in experimental animal models [28,37]. However, it remains to be determined whether maternal diabetes reduces Pax3 protein levels early enough during embryogenesis or whether reduction of *Pax3* expression in diabetes-exposed mice embryos can cause hydrocephalus.

### 4.3. Congenital Hydrocephalus in Patients

Similar to our mouse study, cases of hydrocephalus have been reported in some Waardenburg syndrome patients with various PAX3 heterozygous mutations [12,13]. While other Waardenburg patients with hydrocephalus can carry chromosomal inversions and insertions that do not affect the coding sequence of the PAX3 gene, those with mutations in the regulatory gene(s) for PAX3 [38], the PAX3 promoter, or the 5′ untranslated region of PAX3 [39] are more likely to exhibit phenotypes. Although PAX3 mutations are more usually associated with Waardenburg syndrome and as a cause of neural tube defects, craniofacial-deafness-hand syndrome, alveolar rhabdomyosarcoma, and congenital hearing loss, there is a low prevalence of hydrocephalus reported within PAX3 mutant patients [12,13]. The lethal nature of some homozygous PAX3 mutations [40,41] has largely eradicated the possibility of homozygous mutant survival ,and heterozygous mutant mouse models may provide more clinically relevant models in which to address human disease associated with a given gene mutation. Interestingly, our observation that simultaneous *Pax7* heterozygous mutation increases the frequency of hydrocephalus may imply that mutation or polymorphism of PAX7, leading to loss of function or reduction of PAX7, might be an important genetic contributory factor to the etiology of hydrocephalus. In this regard, the screening for mutation or polymorphism of the PAX7 gene might be a useful addition to evaluate hydrocephalic risk among patients with identified PAX3 mutations.

## 5. Conclusions

Given the clinical observation that some patients with PAX3 mutations are hydrocephalic [12,13], our characterization of the *Pax3* conditional and compound haploinsufficient mutant mouse models has provided an initial molecular basis to begin to understand how genetic, and possibly environmental, factors could affect expression of Pax3 and/or Pax7, and impact the prevalence of congenital hydrocephalus. Taken together, these data suggest that PAX3 haploinsufficiency is a likely risk factor associated with the pathogenesis of congenital hydrocephalus in patients, as well as in animal models.
